# Understanding Genetic Regulation of Sex Differentiation in Hermaphroditic Fish

**DOI:** 10.3390/ani15020119

**Published:** 2025-01-07

**Authors:** Junchao Fang, Guanglve Li, Wenyin Luo, Qiaomu Hu

**Affiliations:** 1 Yangtze River Fisheries Research Institute, Chinese Academy of Fishery Sciences, Wuhan 430223, China; 15119438380@163.com (J.F.); 13659644863@163.com (G.L.); 2 State Key Laboratory of Mariculture Biobreeding and Sustainable Goods, Yellow Sea Fisheries Research Institute, Chinese Academy of Fishery Sciences, Qingdao 266071, China; ioop4916@163.com

**Keywords:** hermaphroditic fish, sex-determining genes, mechanisms of sex differentiation, sexual transition processes, histological alterations

## Abstract

As a fundamental taxonomic group within vertebrates, fish represent an invaluable resource for investigating the mechanisms underlying sex determination and differentiation owing to their extensive geographical distribution and rich biodiversity. Within this biological cohort, the processes of sex determination and differentiation are intricately governed by both genetic factors and the complex interplay of environmental cues. While variations in external environmental factors, particularly temperature, can exert a modulatory influence on sex differentiation in fish to a limited degree, genetic factors remain the primary determinants of sexual traits. Hermaphroditic fish display three distinct types of sexual transitions: protandry (male to female), protogyny (female-to-male), bidirectional sex change (both directions serially). These fish, characterized by their unique reproductive strategies and sexual plasticity, serve as exemplary natural models for elucidating the mechanisms of sex differentiation and sexual transitions in fish. The present review delves into the histological dynamics during gonadal development across three types of sequential hermaphroditic fish, meticulously delineating the pivotal characteristics at each stage, from the inception of primordial gonads to sexual specialization. Furthermore, it examines the regulatory genes and associated signaling pathways that orchestrate sex determination and differentiation. By systematically synthesizing these research advancements, this paper endeavors to offer a comprehensive and profound insight into the intricate mechanisms governing sex differentiation in sequential hermaphroditic fish.

## Simple Summary

This article provides a comprehensive review of the histological changes and key characteristics observed during gonadal development in three types of sequential hermaphrodite fish and further elucidates the associated regulatory genes and signaling pathways involved. The aim is to enhance our understanding of the complex mechanisms underlying sex differentiation in sequential hermaphrodite fish, thereby offering novel insights into research on sex determination. This article explicitly states that the regulatory mechanisms of most sex-related genes are similar across different sequential hermaphrodite fish species; however, a minority of genes demonstrate distinct regulatory patterns among diverse species. Consequently, future studies should integrate multiple levels of regulatory genes, environmental factors, and fine-tuned regulation of the neuroendocrine system to further explore these regulatory mechanisms.

## 1. Introduction

Fish are the lowest vertebrates, and their sex determination is determined by two factors: environmental sex determination (ESD) and genetic sex determination (GSD) [1]. The ESD of fish can be affected by external conditions, such as temperature, photoperiod, pH levels, and hormones. These factors directly impact the sex differentiation of fish, with certain species being more likely to develop into males or females under specific water temperatures. In contrast, GSD is related to sex chromosome genes or sex-linked genes on multiple chromosomes, which play a role in determining the sex characteristics of fish. Many fish species are capable of sex change during their life cycle. For example, in the hermaphroditic clownfish (*Amphiprion clarkii*), when only the female is removed, the largest male in the group will transition into a female to ensure the continuation of the population [2,3]. Although sex change in *A. clarkii* is primarily driven by ESD, it is accompanied by changes in the expression of multilevel female or male sex-linked genes on chromosomes (which will be introduced later) [3], demonstrating the synergistic effect of ESD and GSD [4]. Furthermore, most fish species exhibit sexual size dimorphism (SSD), which refers to significant differences in growth rates between males and females [5]. For instance, female European seabass (*Dicentrarchus labrax*) grow approximately 30% faster than males [6], while male Nile tilapia (*Oreochromis niloticus*) grow about 40% faster than females [7]. Therefore, in-depth research on the regulatory mechanisms of genes and hormones related to sex differentiation in fish can help to artificially control their sex, thereby improving the efficiency of fish farming and production.

To understand the process of sex determination and differentiation in fish, we also need to pay attention to their reproductive modes. Fish reproductive modes can be broadly classified into three types: parthenogenesis, gonochorism, and hermaphroditism [8]. The reproductive patterns of some fish species and the types of sex change in hermaphroditic fish are shown in Table 1.

Firstly, unisexual reproduction in fish is unique as they reproduce without the fusion of gametes from two sexes, instead developing directly from a single-sex cell into a new individual. This reproductive mode typically occurs in harsh environments or when population numbers are low and serves as an adaptive strategy for survival and reproduction. Only a very few fish species, such as the Amazon molly (*Poecilia formosa*) [22,23], are capable of parthenogenesis. Secondly, gonochorism is the most common reproductive mode in fish. After sex differentiation, these fish possess only one type of gonad, either testes or ovaries. Therefore, they need to combine sex cells with the opposite sex to form fertilized eggs, which then develop into new individuals. Additionally, different gonochoristic fish species may have different sex determination systems, such as XY or ZW. Finally, in hermaphroditic reproductive modes, fish individuals undergo sex change at some point in their life cycle, allowing the same fish to serve as either the male or female parent in reproduction. It is worth mentioning that not all hermaphroditic fish require sex cells from the opposite sex for reproduction, as very few species, such as the Mangrove Rivulus Fish (*Kryptolebias marmoratus*) [24], are capable of self-fertilization. Although hermaphroditism is relatively rare, it provides us with valuable opportunities to study sex determination and differentiation in vertebrates, as their genome remains unchanged while their phenotype changes during sex change. Research on the mechanisms of sex change in these fish can provide a more comprehensive understanding of the influence of molecular factors involved in sex determination on sex differentiation [25]. Therefore, this article will discuss four parts: the gonadal developmental changes in three types of hermaphroditic fish, the regulatory mechanisms of sex determination and differentiation in fish, and some sex-linked genes in order to better understand the role and mechanisms of sex determination-related genes in hermaphroditic fish.

## 2. Changes in Gonadal Development in Hermaphroditic Fish

Hermaphroditic fish often exhibit adaptive reproductive strategies that enable them to withstand a broad spectrum of environmental perturbations. They can be classified into three distinct categories based on the sequence of sexual transitions: protandry (male to female), protogyny (female-to-male), and bidirectional sex change (both directions serially) [26]. The occurrence of hermaphroditism among fish is not common, as approximately 1% of vertebrate species that possess this trait belong to the fish kingdom [27,28]. This proportion is even higher among scleractinian fish, accounting for approximately 2% of the total. These fish, possessing both male and female reproductive organs, are predominantly distributed in temperate and tropical aquatic environments, spanning more than 20 taxonomic families within nine different orders [29].

### 2.1. Protandrous Hermaphrodite Fish

**Figure 1:**
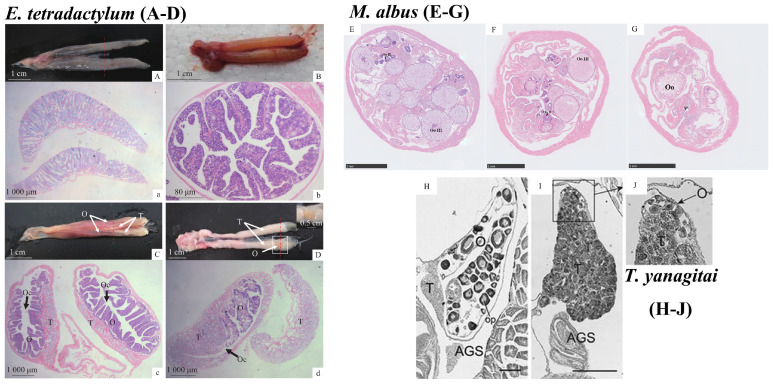
The gonadal tissue structure of three hermaphroditic fish species. (**A**–**D**): *E. tetradactylum* (taken from Lan et al.) [10]; (**E**–**G**): *M. albus*; (**H**–**J**): *T. yanagitai* (taken from Makoto et al.) [19]; (**A**) Anatomy of the testis; (**a**) Cross section of testis; (**B**) Anatomy of the ovary; (**b**) Cross section of the ovary; (**C**,**D**) Anatomy of the transitional gonads; (**c**,**d**) Cross section of the transitional gonads; (**E**) Pre-intersexual development; (**F**) Middle intersexual development; (**G**) Late intersexual development; (**H**) Female, (**I**,**J**) male; Oo II: II phase Oocytes; Oo III: III phase Oocytes; Oo: Oocytes; Oc: Ovarian cavity; PSC: primary spermatocyte; AGS accessory gonadal structure; op: oocytes in perinucleolus stage; (**A**–**D**) Bar 1 mm; (**H**–**J**) Bar 0.5 mm.

*A. australis*, *A. schlegeli*, *R. sarba*, and *E. tetradactylum* of the horse mackerel family belong to a special group of fish: protandrous hermaphrodite fish. The process of gonadal changes in these fish is unique and interesting (Figure 1A–D). At the juvenile stage, these fish exhibit male characteristics, with their gonads containing non-functional spermathecal tissue and immature ovaries consisting mainly of primary oocytes. The gonads evolve into the mature male stage, which is characterized by the presence of immature ovaries containing pre-vitellogenic oocytes in addition to spermathecae at various developmental stages. At this stage, the fish still maintain male reproductive capacity, but the changes within the gonads signal an impending sex transition. Subsequently, these fish enter a phase of sexual reversal, in which the spermathecal tissues begin to progressively degenerate and release large quantities of free spermatozoa. Meanwhile, ovarian tissue continues to develop and fill with oocytes in the pre-oviposition stage. This marks a critical period of transition, during which the fish’s reproductive organs undergo a complete transition from male to female characteristics. Finally, these fish enter the mature female stage. At this point, the ovaries are filled with oocytes at various developmental stages, while the spermathecae are severely degenerated and atrophied. The process of sex reversal is then complete, and these fish become functional females [9,10]. It is essential to note that the parthenogenetic gonads of protandrous fish have a characteristic feature: the coexistence of progressively degenerated spermathecal tissue and oocytes in the vitellogenic stage. This feature can be an important criterion for identifying whether a hermaphroditic fish is protandrous [30].

During sexual reversal in hermaphroditic fish, the development of the gonads undergoes a series of remarkable histological and morphological changes. Taking *E. tetradactylum* as an example, we can observe the different stages of these changes in detail. In the early stages of sexual reversal, the gonads of *E. tetradactylum* show a grayish-white flattened banded appearance, similar to typical male spermathecae. Examining the tissue sections, we observe elongated pike-shaped gonads with a large empty ovarian chamber in the center. This chamber appears to divide the gonads into two parts: the spermatheca and the ovary. At this stage, one or two layers of oogonia are present at the junction of the seminal and ovarian cavities. Although they are bounded by connective tissue, the boundary is not clearly defined. At this stage, the ovarian cavity is yet to be fully formed by the egg-laying plate, while the spermathecal portion resembles the typical primordial spermathecae, containing all levels of spermatogenic cells in the spermathecal lobules and a relatively large number of mature spermatozoa in the spermathecal lumen. In the middle stage, the appearance of the gonads becomes thickly banded or rod-shaped, with a creamy-white spermathecal portion on the medial side of the dorsum and a fleshy-red ovarian portion on the lateral side of both abdomens. Tissue sections reveal that the gonads are oval in shape, with the peritoneal portion of the outer layer thickened. At this stage, the ovaries start developing toward the spermathecae, increasing in size. Simultaneously, the connective tissue between the spermathecae and ovaries thickens, creating a more distinct boundary between the two. Oogonia progress to phase II, and the spawning plate takes on a fully formed, bifurcated, finger-like shape. Conversely, the density of mature spermatozoa decreases in the seminiferous lobules of the ovarian part of the spermathecae. The connective tissue between the lobules thickens, and spermatocytes at the margins begin to degenerate, leading to the formation of unclear and blurred structures. During late sexual reversal, the gonads develop an orange rod-like appearance, similar to that of typical female gonads. Tissue sections indicate that the gonads become ovoid and mostly transition into ovarian tissue. At this stage, the ovipositor plate is well-developed and occupies the ovarian cavity. In contrast, the gonad located next to the early spermathecae has only a small layer of spermathecal structures on the edges, and the spermatozoa within these spermathecal lobules have long since degenerated [10].

### 2.2. Protogynous Hermaphrodite Fish

The protogynous hermaphrodite fish occupy a prominent position among the hermaphroditic fish. Grouper fish, such as *E. coioides*, *E. akaara*, and *E. bruneus,* represent this type among marine fish, while *M. albus* represents it among freshwater fish. The process of sex development in this group of fish is unique in that they exhibit female characteristics at the juvenile stage, and when the female matures and completes spawning or under certain environmental conditions, her ovarian tissue gradually degenerates while the spermathecal tissue begins to develop, leading to the sex reversal stage. Eventually, the ovaries completely degenerate, and the female fish undergo a transition into functional male fish [12,13,14,31]. During the process of sex reversal, protogynous hermaphrodite fish exhibit some distinctive features, such as the coexistence of degenerated mature oocytes and proliferating spermatocytes, as well as the residual ovarian cavity in the spermatheca after sex reversal. These features provide us with an important basis for judging the process of their sex reversal [8].

In *M. albus*, for example, after reaching sexual maturity and spawning for the first time, oocytes start to degenerate and undergo sexual reversal. This process can be divided into three stages based on the histological and morphological features of gonad development: early intersexual, middle intersexual, and late intersexual (Figure 1E–G) [32]. In the early intersexual stage (Figure 1E), the gonads appear pale orange with visible yellowish-white oocytes. During this stage, the oocytes start decreasing, with the presence of abortive oocytes in various phases, while the germinal folds thicken, and primitive spermatogonia begin to form. As the middle intersexual stage (Figure 1F) progresses, the gonadal appearance changes to a creamy-white streak, and the oocytes are no longer visible. Tissue sections show the presence of oocytes in different phases, more empty spaces in the cystic cavity, and further widening and thickening of the gonadal folds, which are lined with more spermatogonia. Finally, in the late intersexual stage (Figure 1G), the gonads still have a creamy-white striped appearance, but with black patches resembling residual degenerated oocytes [33]. At this point, the number of oocytes is minimal, the germinal folds fill the cystic cavity, and a large number of spermatogonia are present inside, some of which develop into primary spermatocytes.

### 2.3. Bidirectional Sex-Changing Fish

Bidirectional sex-changing fish represent a unique type of fish that possess both functional gonadal tissues and can adjust their gender expression in response to changes in their social status within the population. This type of fish is uncommon in nature, with 10 species known from five families, including *G. erythrospilus* and *L. dalli* from the Gobiidae family and *K. marmoratus* from the Killifish family [14,17,18].

In deep-water gobiid fish (*T. yanagitai*) (Figure 1H–J), both spermathecal and ovarian tissues separated by connective tissues are present in the gonads, along with auxiliary gonadal structures (AGS). In mature females, most of the gonad comprises ovaries and AGS containing oocytes at various developmental stages, while the spermathecae occupy a small portion filled with spermatogonia. Conversely, in mature males, most of the gonads comprise the spermatheca and AGS, which are filled with fully developed sperms. The ovaries occupy only a small portion and contain few oocytes. It is important to note that *T. yanagitai* practices polygynous mating, with all individuals in the population being male except for the largest individual. This allows it to adjust its gender expression based on its social status within the population [19]. Although Trimma fish, as well as fish in the Snappers family like *A. schlegeli* (male before female) and red seabream (*Pagrus pagrus*) (female before male), are also separated by connective tissues, they do not synchronize the maturation of males and females. This suggests that the connective tissue barrier may be necessary but not sufficient for the simultaneous maturation of the spermatheca and ovary [14].

## 3. Regulatory Mechanisms of Sex Determination and Differentiation in Fish

The sex determination mechanism of scleractinian fish is a complex and diverse area. They have a rich variety of sex determination system types, which are mainly categorized into the following five types [34]: XX/XY male heterogametic type, which is the sex chromosome type of most fish, and *O. niloticus* [35] and Japanese medaka (*Oryzias latipes*) [36] are the representatives belonging to this type of sex determination. ZW/ZZ female heterogametic type: contrary to the XX/XY type, in the ZW/ZZ type, the sex chromosome combination is ZW for females and ZZ for males. Blue tilapia (*Oreochromis aureus*) [37] and Chinese tongue sole (*Cynoglossus semilaevis*) [38] are examples of fish with this type of sex determination. ZZ/ZO and XX/XO Chromosome Deletion Heterozygous: This type of chromosome combination is characterized by the absence of a sex chromosome. For example, the shortjaw tapertail anchovy (*Coilia brachygnathus*) [39] belongs to this type. X1X1X2X2/X1X2Y complex sex chromosome type: in this type, females or males have more than two sex chromosomes, as in rock bream (*Oplegnathus fasciatus*) [40]. Autosomal type: sex determination in some fish does not depend on distinct sex chromosomes, but may be dominated by genes on autosomes, e.g., zebrafish (*Danio rerio*) [41].

**Figure 2:**
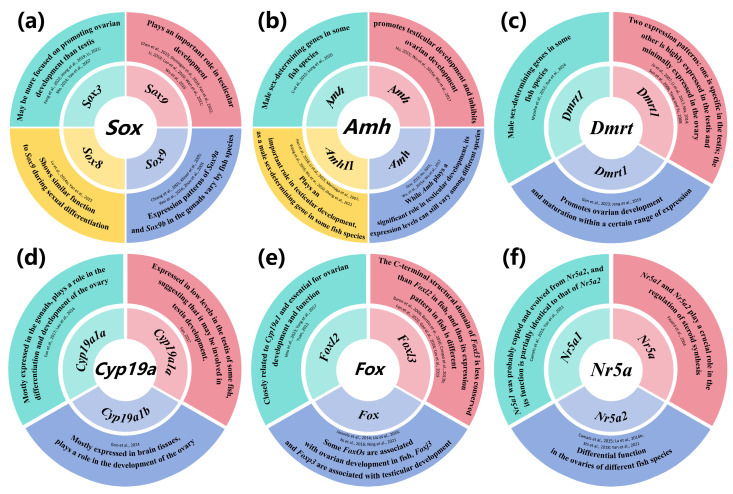
Potential functions of sex genes ((**a**) *Sox* [11,12,45,46,47,48,49,50,51,52,53,54,55,56,57,58,59,60], (**b**) *Amh* [35,61,62,63,64,65,66,67,68,69,70], (**c**) *Dmrt* [34,36,56,71,72,73,74,75,76], (**d**) *Cyp19a* [77,78,79,80], (**e**) *Foxl* [50,81,82,83,84,85,86,87,88,89,90,91,92] and (**f**) *Nr5a* [93,94,95,96,97]) in fish sex differentiation and development.

Previous studies have shown that the genes regulating sex determination and differentiation in fish can be divided into four levels [42]. The first level consists of sex-determining dominant genes, which control the differentiation process of gonads toward males or females. Among these genes, male-determining genes are predominant, as shown in Table 2. The second level comprises upstream regulatory genes of sex differentiation, such as *Sox9*, *Amh*, *Dmrt1*, and *foxl2*. These genes serve as initiators of gonadal differentiation in fish and have the ability to directly or indirectly regulate the expression of downstream sex steroid genes. The third level involves midstream regulatory genes, primarily responsible for synthesizing and releasing steroid hormones, such as the genes *Cyp19a* and *Cyp11b*. These genes play crucial roles in sex differentiation. The final level includes downstream steroid hormone synthesis-related genes, such as androgen receptor genes (*ar*) and estrogen receptor genes (*er*), which respond to signals from upstream and midstream genes and regulate the formation and maintenance of sex characteristics. Currently, researchers have identified numerous sex-determining genes in fish, which can be broadly classified into two groups: transcription factors and *TGF-β* signaling pathways. The transcription factor category includes DM structural domain genes (e.g., *Dmrt1*) and *Sox* protein family members (e.g., *Sox3*, *Sox8*, and *Sox9*), while the *TGF-β* signaling pathway category includes genes such as *Amh*, *AmhrII*, and *Gsdfy*. These findings suggest that the fish sex-determining gene network that regulates sex differentiation and gonadal development may be conserved. It is noteworthy that some sex-related genes in specific fish species have unique origins and functions. For instance, the *Sdy* gene of rainbow trout (*Oncorhynchus mykiss*) originates from a duplicated copy of an immunity gene [43], while *hsd17b1* of yellowtail (*Seriola quinqueradiata*) is a steroid hormone convertase gene [44]. Since this study focuses on sex-related genes in hermaphroditic fish, the subsequent sections will briefly discuss female and male sex-related genes separately and are summarized in Figure 2. These genes play vital roles in sex determination and differentiation in hermaphroditic fish, and their interactions and regulatory mechanisms collectively ensure the accurate formation and maintenance of sex characteristics in fish.

## 4. Male Sex-Related Genes

Within the research scope of Topic 3, we have uncovered the existence of various sex determination systems and sex-determining genes, among which male sex-determining genes dominate, while *Hsd17b1* stands out as distinct. Notably, in the gonochoristic *S. lanzhouensis* species with *AmhrIIY* as the sex-determining gene, an XY genotype “intersex” phenomenon was serendipitously discovered, wherein the gonad concurrently possesses mature ovarian and testicular tissues, enabling self-fertilization [61,108]. This finding further highlights the complexity of sex gene regulatory mechanisms in fish. Therefore, while delving deeply into the regulatory mechanisms of sex-related genes in intersex fish, we must also consider their potential roles in gonochoristic fish species.

### 4.1. Sry-Related High Mobility Group Box

The *Sox* gene family serves as a key transcriptional regulator in vertebrates and invertebrates. Their members encode proteins with unique high mobility group box (HMG-box) structural domains, enabling them to bind tightly to DNA and regulate gene expression [109]. These genes are crucial for sex determination and gonadal development. The earliest identified *Sox* gene is the sex-determining region of the Y chromosome (*Sry*) gene located on the Y chromosome. It plays a vital role in male sex determination in mammals and indicates that the sex determination mechanism is evolutionarily conserved [20,110]. Over 40 different *Sox* genes have been identified in fish, amphibians, reptiles, birds, and mammals. These genes can be further categorized into 11 subfamilies from A-K, each with unique expression patterns and functions. They regulate embryonic and gonadal development and also impact the growth and development of the heart, blood vessels, and bones [21].

In scleractinian fish, genes such as *Sox3*, *Sox8,* and *Sox9* are closely associated with gonadal development and sex determination. Specifically, *Sox8* and *Sox9*, which have similar roles in sex differentiation, boost the expression of *Amh* by forming a complex with steroidogenic factor-1 (*SF-1*) and co-activating the *Amh* promoter [11]. While *Sox8* can substitute *Sox9* in some functions, *Sox9* plays a more central role in determining sex and the development of reproductive organs, particularly in the development of sperm cells [45]. Numerous studies have demonstrated that there is a significant difference in the expression of *Sox9* between the sexes in fish reproductive organs, with higher expression levels usually found in males compared to females. This disparity has been confirmed in various fish species, such as the gonochoristic *S. argus* [46] and *P. olivaceus* [47], the protandrous Barramundi (*Lates calcarifer*) [48], and the protogynous *E. coioides* [12]. In the hermaphroditic *M. albus* and *E. coioides*, the expression level of *Sox9* increases during the process of sex reversal [49,50]. It has been observed that the expression of *Sox9* mRNA significantly increased after female *E. coioides* were subjected to 17α-methyltestosterone (17α-MT) treatment, leading to sex reversal [12]. This finding emphasizes the crucial role of *Sox9* in male sex determination and testicular development. It is important to note that there are two forms of *Sox9*, *Sox9a* and *Sox9b*, in scleractinian fish. Their expression patterns in gonads may vary depending on the fish species. For example, in *D. rerio*, *Sox9a* is mainly expressed in the spermatheca, while *Sox9b* is expressed in the oocyte [51,52]; however, in *O. niloticus*, the opposite is true [53]. In *M. albus*, *Sox9a* exists in two copy forms, *Sox9a1* and *Sox9a2*, and is expressed in the testis, ovary, and ovotestis [54]. The differences in the expression patterns of these species suggest that they have different roles in sex determination and gonadal development. Mutations in the *Sox9* gene can result in phenomena such as retarded gonadal development and sex reversal. For instance, mutations in the *Sox9b* gene in *O. niloticus* cause delayed development of its spermathecae, while mutations in the *Sox9* gene in *O. latipes* may lead to a transition from male to female [55]. These studies further confirm the critical role of *Sox9* in sex determination and gonadal development.

Remarkably, *Sox3* plays a crucial role in the mechanism of sex determination; it has the highest sequence similarity to *Sry*. In XX/XY sex-determining organisms, *Sox3* is located on the X chromosome, a position that further emphasizes its potential influence on the sex determination process [111]. Further evidence supporting *Sox3’s* pivotal role in sex determination comes from the fact that it is even present in *O. dancena* as the male sex gene *Sox3Y* on the Y chromosome [56]. A number of significant discoveries have been achieved recently, despite the fact that there have been comparatively few studies on fish *Sox3*. For example, *Sox3* expression in the ovaries was considerably higher than that in the spermatheca of black rockfish (*Sebastes schlegeli*) [56], *E. coioides* [57], and *C. vrolikii* [58]. After interfering with *Cv-Sox3*, the oocyte growth rate of *C. vrolikii* decreased, and the expression of the ovarian bias-related genes *Cyp19a* and *Foxl2* decreased significantly. Meanwhile, the expression of testicular bias-related genes *Dmrt1, Sox9*, *Amh,* and *Sox8* increased. These studies suggest that *Sox3* plays an important role in ovarian development in fish. *A. schlegeli*, on the other hand, had the exact opposite condition [112], indicating that various fish species may have different *Sox3* expression patterns. In addition, studies in bearded catfish (*Clarias batrachus*) [113] have shown that *Sox3* is associated with the development of the male testis, further adding to the complexity of the role of *Sox3* in sex determination mechanisms. *Sox3* seems to be more inclined to promote ovarian development compared to *Sox9*. For instance, studies on *D. rerio* and *O. niloticus* found that reducing the activity of *Sox3* led to a decrease in the number of eggs [59]. Furthermore, in *D. rerio* studies, *Sox3* was found to combine and activate the expression of *Cyp19a1a*, which promotes *17β-estradiol (E2)* synthesis and finally inhibits apoptosis in ovarian cells [60], indicating the significance of *Sox3* in ovarian development. In conclusion, *Sox3* plays an important role in ovarian and testicular development; however, *Sox3* may be more focused on promoting ovarian development than testicular development.

### 4.2. Transforming Growth Factor-β

The transforming growth factor-β superfamily is a large family containing at least 30 related ligands [114]. These ligands play important roles in various physiological processes in organisms. Some important members of this family include bone morphogenetic proteins (BMPs), growth and differentiation factors (GDFs), activins, and anti-Müllerian tube hormone (Amh) [115]. Among these members, the *Amh* and *AmhII* genes have received much attention for their critical roles in sex determination and gonadal development.

*Amh* is produced primarily by supporting cells and is commonly found in vertebrates. Although scleractinian fish do not have Müllerian ducts, they still possess the *Amh* gene. Numerous studies have shown that *Amh* plays a crucial role in male sex determination and gonadal development. For example, *O. niloticus*, Hatcher’s toothfish (*Odontesthes hatcheri*), and northern pike (*Esox lucius*), despite their distant affinities, have the sex-determining gene *Amhy* located on the Y chromosome [63]. Further research has indicated that *Amh* is positively associated with *Sox9* expression, which aligns with the *Amh-Sox9* male sex gene expression pathway [49]. Strong evidence is also provided by the expression pattern of *Amh* during sex reversal in fish, where females mature first in hermaphrodites. In *M. albus*, *Amh* expression is low in the female gonad, but is significantly elevated upon entry into intersexual stage II, maintaining high gene expression from intersexual stage II through the male stage [64]. Similarly, in *E. coioides*, *Amh* is consistently expressed in cells surrounding type A spermatogonia-like cells, and its expression is enhanced as spermatogonia develop during the interphase and continue through the male stage [65]. In contrast, in protandrous hermaphrodite fish such as *A. schlegeli*, *Amh* is expressed in spermathecal support cells and limits the size of the ovary by inhibiting its proliferation [66]. These findings suggest that *Amh* not only contributes to the development of the spermatheca, but also inhibits the development of the ovary. Regarding the specific mechanism of *Amh*’s action, it was shown that it may inhibit ovarian development in female fish by downregulating the expression of *Cyp19a1a*. In addition, *Amh* may act together with various genes, such as *SF-1* and *Sox9,* to induce and promote the development of spermatheca in male fish [42]. While *Amh* plays a significant role in spermatheca development, its expression levels can vary among different species. For instance, in adult pond loach (*Misgurnus anguillicaudatus*), *Amh* expression is higher in the ovary than in the spermatheca, whereas in adult Large-scale Loach (*Paramisgurnus dabryanus*), the difference in expression between the spermatheca and the ovary is relatively small [67].

*AmhrII* acts as a type II receptor for *Amh* and induces degeneration of the Müllerian duct in male mammals. In fish, *AmhrII* also plays a crucial role in sex determination and gonadal development. For instance, in Tiger Puffer (*Takifugu rubripes*), the sex is determined by the SNP locus of the *AmhrII* gene, with testicular *AmhrII* transcript levels significantly higher than those of the ovary in both *A. schlegeli* and *E. coioides* after gonadal differentiation [68,69]. In *O. niloticus*, knockout of *AmhrII* resulted in sex reversal from male to female in all individuals, along with an increase in *Cyp191a* expression and estrogen levels [32]. Even in *O. latipes*, where the sex-determining gene is *Dmy* (*Dmrt1bY*), mutations in *AmhrII* cause hyperproliferation of germ cells, sex reversal from XY males to females, and expression of gonadal somatic cell aromatase [70]. These findings indicate that *AmhrII* not only plays a crucial role in male sex determination and differentiation, but also inhibits aromatase and estrogen expression. Although the *Amh-Smads-Cyp19a1a* signaling pathway has been demonstrated in various mammals and *E. coioides*, there is still insufficient direct evidence regarding the regulation of *Cyp19a* by the *Amh/AmhrII* receptor system [116]. Further research in this area is necessary to gain a deeper understanding of the complex mechanisms involving these genes in sex determination and gonadal development.

### 4.3. Doublesex and Mab-3 Related Transcription Factor

The *Dmrt* gene family is widespread in vertebrates and includes numerous members, such as *Dmrt1*, *Dmrt8,* and *Dmrt2b*. These genes are important for sex determination, embryonic development, and muscle development [117]. *Dmrt1* is particularly essential for male sex determination and spermatophore development and has been identified as a sex-determining gene in fish species like *O. latipes* [36] and *C. semilaevis* [38]. The expression pattern of *Dmrt1* can be divided into two types [56]. The first type is specifically expressed in the spermatheca, as seen in species such as *P. olivaceus* [71], adult *C. semilaevis* [72], and *O. niloticus* [73]. The second type is highly expressed in the spermatheca and minimally expressed in the ovary, as observed in species such as honeycomb grouper (*Epinephelus merra*) and *O. mykiss* [74]. Similarly, *Dmrt1* expression is higher in the spermathecae than in the ovaries of hermaphrodite fish such as *M. albus* and *A. schlegeli* [118,119], and knockdown of *Dmrt1* in *A. schlegeli* leads to a reduction in the number of germ cells in the spermatheca, thereby inducing its sex reversal from male to female [120]. *Dmrt1* accelerates spermatogenesis by stimulating the *Rec8* gene to put spermatogonia into the meiotic process; implantation of *Dmrt1* and *Rec8* expression plasmids into the testis of *E. akaara* induces sperm maturation in vivo [121]. In addition, implantation of *Dmrt1* from Chinese medaka (*Oryzias sinensis*) into *O. latipes* resulted in the conversion of XX-type *O. latipes* from females to males [122]. These studies demonstrate that *Dmrt1* plays a critical role in inducing testicular development and sex reversal. In addition, *Dmrt1* was shown to repress *O. niloticus* basal *Cyp19a1a* transcription and *SF-1*-activated *Cyp19a1a* transcription [123], which further confirms the centrality of *Dmrt1* in male sex determination and testicular development.

Although the function of *Dmrt1* has been better understood, a recent study found that *Dmrt1* may play a role in oogenesis in the Japanese eel (*Anguilla japonica*) [75], and that *Dmrt1a* regulates *Foxl2*- and *Nr5a1*- induced activation and synergistic effects of *Cyp19a1a* transcription in a biphasic manner at different doses. A high dose (100–200 ng) of *Dmrt1a* can stop inhibiting or even promote *Cyp19a1a* transcription and *E2* production, thus promoting the development and maturation of the ovary in *M. albus* and other vertebrates [76]. This suggests that the functions of *Dmrt1* are more complex and diverse than previously understood. Hence, further experiments are crucial to explore its functions and regulatory mechanisms in depth.

## 5. Female Sex-Related Genes

Female sex-related genes also play a crucial role in gonadal differentiation and development in fish. Among them, the *Cyp19a* gene encodes cytochrome P450 aromatase, which is a key enzyme for converting androgens into estrogens. Additionally, genes such as *Foxl2* in the Fox gene family are involved in the regulation of ovarian function in fish and interact closely with *Cyp19a1* to promote ovarian development. *Nr5a* genes, such as *Nr5a1* and *Nr5a2*, in the nuclear receptor superfamily also play important roles in regulating aromatase expression and gonadal development. These genes jointly maintain the normal development and function of female gonads through a complex regulatory network.

### 5.1. Cytochrome P450 Family 19 Subfamily A Member 1

*Cyp19a* plays an important role in fish and other vertebrates mainly by encoding cytochrome P450 aromatase (*P450arom*), a key enzyme for converting androgens to estrogens. There are two aromatase genes in scleractinian fish [124]: *Cyp19a1a* (also referred to as *Cyp19a* or *Cyp19a1*), which is the aromatase gene of the gonadal gland, and *Cyp19a1b* (also referred to as *Cyp19b* or *Cyp19a2*), which is the aromatase gene of the brain type.

*Cyp19a1a* is mostly expressed in the gonads, where it is essential for the production of *E2* and plays a role in the differentiation and development of the ovary [77,78]. The expression of *M. albus Cyp19a1a* is stimulated by gonadotropin via the Adenosine-3′,5′-cyclic monophosphate (cAMP pathway) in the ovary but not in the ovotestis or testis. Meanwhile, the methylation level of the *Ad4BP/SF-1* protein locus was negatively correlated with the expression of *Cyp19a1a*, and the expression of *Cyp19a1a* gradually decreased during the sex reversal process [125,126]. Similarly, *LRH-1* from *E. coioides*, which combines with *Cyp19a1a* in vivo, is highly expressed in the ovaries during the yolk-forming stage and is significantly reduced during the maturation stage [93]. *Cyp19a1a* is closely related to estrogen synthesis, which is reduced when *Cyp19a1a* expression decreases, leading to a female-to-male transition in scleractinian fish. The above studies revealed the important role of *Cyp19a1a* expression in sex reversal in scleractinian fish and showed that its expression is biased toward females. In addition, *M. albus* gonads that had *Cyp19a1a* knocked down did not develop into ovaries, highlighting the crucial role of this gene in ovarian development [81].

*Cyp19a1b*, primarily expressed in brain tissues, regulates ovarian differentiation, development, maturation, and spawning by controlling not only the synthesis of estrogen through estrogen receptors but also the synthesis and secretion of follicle-stimulating hormone (FSH) and luteinizing hormone (LH) by secretory cells [79]. In the brains of *O. latipes* [127] and the hermaphrodite *T. bifasciatum* [15], the expression of *Cyp19a1b* was significantly higher in females than in males; however, there was no significant difference between the two expressions in the brains of rare minnow (*Gobiocypris rarus*) [128], *D. rerio* [129], blue gourami (*Trichogaster trichopterus*) [130], and largemouth bass (*Micropterus salmoides*) [131]. Although the pattern of brain *Cyp19a1b* expression is not entirely consistent across fish species, it has been shown that *Cyp19a1b* is involved in the regulation of *E2* synthesis through the brain-pituitary-gonadal axis (B-P-G axis), as well as the upregulation of *Cyp19a1b* expression induced by brain *E2* (neuroestrogen) [131,132,133]. Hence, these two aromatase genes play important regulatory roles in the differentiation and development of fish gonads. It is also interesting to note that *Cyp19a1a* is poorly expressed in the spermathecae of some fish, suggesting that aromatase genes may play a role in the development of fish spermathecae. This is further supported by the finding that in male *A. schlegeli*, low doses of *E2* enhanced spermathecal development [80].

### 5.2. Winged Helix/Forkhead Transcription Factor

The *Fox* gene family is a large and important group of genes with 19 types *(Foxa-Foxs)* [82]. It has been shown that many *Fox* genes are involved in gonadal regulation and development; for example, *Foxl2* and various *FoxO* genes have been reported to be involved in the regulation of ovarian function in fish [82,83], while *Foxj3* and *Foxp3* have been associated with spermatogenesis and testicular function in mice [84,85], among which *Foxl2* plays a key role in female sex determination and differentiation in vertebrates. Its expression is primarily confined to ovarian follicular layer cells, exhibiting significant sex differences, as confirmed in a variety of fish and other vertebrates, including *M. albus* [81,86].

Numerous studies have shown that there is an antagonism between *Foxl2* and *Dmrt1* during sex differentiation in vertebrates [134]. In scleractinian fish, *Foxl2* has two isoforms, *Foxl2a* and *Foxl2b* (also known as *Foxl3* in a variety of scleractinian fish [87,88,89]). These two isoforms, although structurally similar, may differ in their functional expression and regulatory mechanisms. For example, In the *O. mykiss*, *Foxl2a* and *Foxl2b* were specifically expressed in the ovary, suggesting that they are involved in the regulation of ovarian development [89]. However, in Atlantic salmon (*Salmo salar*), *D. labrax,* and *E. coioides*, *Foxl3* is mainly expressed in the testis, suggesting that it may play a role in spermatheca development, while *Foxl2* and *Foxl3* were expressed in the ovaries of hermaphrodite fish, such as *E. coioides* and *M. albus*, *Foxl3* expression was relatively low and gradually increased with the process of their sex reversal [50,87,90,91]. Hence, *Foxl3* may be involved in the regulation of ovarian and testicular development, but its expression pattern varies in fish due to the fact that the C-terminal structural domain of *Foxl3* is poorly conserved in fish compared to *Foxl2* [88]. In addition, it was found that *Foxl2* closely interacts with *Cyp19a1*, a key gene encoding an aromatase enzyme responsible for converting androgens to estrogens. This process is essential for ovarian development and function [92]. To increase estrogen levels and encourage ovarian development, *Foxl2* can either directly bind to the *Cyp19a1* promoter or, through interaction with *Ad4BP/SF-1 Cyp19a1* expression, stimulate *Cyp19a1* expression [135,136]. The molecular mechanisms underlying ovarian development and sex determination are now better understood in light of this finding. *Foxl2* may regulate ovarian function via the brain-pituitary-gonadal axis (B-P-G axis) in addition to directly regulating *Cyp19a1* expression [137]. This is a complex regulatory network involving the interaction of multiple hormones and genes that work together to maintain normal ovarian function.

### 5.3. Nuclear Receptor Superfamily

The nuclear receptor superfamily is a significant and extensive family of transcription factors, the activation of which depends on ligands. This family comprises 48 members and is crucial for various biological processes, such as metabolism, sex determination, differentiation, and gonadal development and maintenance. Based on sequence comparison and phylogenetic tree construction, the nuclear receptor superfamily was subdivided into seven subfamilies from *Nr0* to *Nr6*. Notably, the *Nr5a* gene, belonging to the orphan nuclear receptor family, plays a vital role in regulating aromatase expression [138].

There are five members of the *Nr5a* subfamily, *Nr5a1-Nr5a5* [94,95,139], of which steroidogenic factor-1 (*Nr5a1*, also known as *SF-1/Ad4BP*) and hepatic receptor homolog-1 (*Nr5a2*, also known as *LRH-1*) play crucial roles in the regulation of steroid synthesis [94]. In mammals, *Nr5a1* is mainly expressed in steroidogenic tissues, whereas *Nr5a2* is mainly expressed in tissues of endodermal origin (e.g., pancreas, liver, and intestines) and gonads [140]. Notably, the interaction of *Nr5a1* with *Foxl2* enhances *Cyp19a1* expression, which has been demonstrated in various scleractinian fish [135,141]. For example, deletion of *Nr5a1* in *O. niloticus* leads to a decrease in *Cyp19a1a* and *Foxl2* expression and serum *E2* levels in XX females, which in turn triggers gonadal hypoplasia and sexual reversal in some fish [142].

Several researchers have identified four *Nr5a* homologs in the *M. albus* genome: *Nr5a1a*, *Nr5a1b*, *Nr5a2*, and *Nr5a* [95]. Their findings indicate that *Nr5a1a*, in combination with *Foxl2*, upregulated *Cyp19a1a* expression in *M. albus*. The other three *Nr5a* homologs did not enhance the impact of *Foxl2* on *Cyp19a1a* promoter activity, either alone or together. In studies on *E. coioides*, the expression of either *Nr5a1a* or *Nr5a2* alone was able to upregulate *Cyp19a1a* transcription, and the effects were superimposed when both were expressed simultaneously, further confirming that *Nr5a1a* and *Nr5a2* upregulate the transcription of *Cyp19a1a* through the same mechanism [93,96]. Regarding the evolution of the *Nr5a2* gene, it has been shown that the chordate ancestor may have first generated the *Nr5a2* and *Nr5a5* genes through duplication of the *Nr5a* gene, followed by further duplication of the *Nr5a2* gene, ultimately leading to the present *Nr5a1* and *Nr5a2* genes. Given their homology, these two genes have some shared functions. For example, in mammalian studies, *Nr5a2* was found to partially replace the function of *Nr5a1* in promoting steroid synthesis [97]. Hence, the *Nr5a* genes play an important role in gonadal development and maintenance. *Nr5a1* and *Nr5a2* are able to act synergistically to regulate ovarian development in females, but the roles of the separate *Nr5a2* genes may differ between ovaries of different fish species. Therefore, further in-depth studies on the function of the *Nr5a2* gene in the gonads are required.

## 6. Conclusions

The regulatory mechanisms of sex-determining genes in fish are exceedingly complex, with those of hermaphroditic fish involving multiple layers of regulatory genes, environmental factors, and fine-tuned modulation of the neuroendocrine system. Despite some advancements in our understanding, numerous unknown regulatory mechanisms remain to be explored. From the literature reviewed, several conclusions can be drawn.

(1) The types of sex determination systems and sex-determining genes in fish are diverse and abundant.
(2) The key genes involved in the sex differentiation process of hermaphroditic fish are relatively conserved; however, their regulatory mechanisms may vary among different species, as exemplified by *Sox3*.
(3) The sex differentiation process in hermaphroditic fish resembles a continuous tug-of-war between pathways related to the two sexes, with the victorious pathway determining the direction of gonadal differentiation.

Under laboratory conditions, *M. albus* originally identified as females from the larval stage were found to partially transition into males by the age of 5 months, and no degenerating oocytes were observed in the testes of these male individuals [143]. It is noteworthy that previous studies have indicated that *M. albus* are almost exclusively female before reaching the age of 2 years. Furthermore, under laboratory conditions, *O. latipes* (XY type) exhibits temperature-dependent sex determination (TSD) [4]. Therefore, in the future, as we strive to gain a deeper understanding of the sex regulatory mechanisms in hermaphroditic fish, it is imperative to consider species from diverse environments in order to better comprehend the potential roles of sex genes in these fascinating organisms.

## Figures and Tables

**Table 1:** Reproductive patterns of some fish species and types of sex change in hermaphroditic fish.

Reproduction Patterns		Exemplar Species	Families	Reference
hermaphroditic	protandry	*Acanthopagms australis*	*Sparidae*	[9]
		*Acanthopagrus schlegeli*	*Sparidae*	[9]
		*Rhabdosargus sarba*	*Sparidae*	[9]
		*Eleutheronema tetradactylum*	*Polynemidae*	[10]
		*Lates calcarifer*	*Latidae*	[11]
	protogyny	*Epinephelus coioides*	*Serranidae*	[12]
		*Epinephelus bruneus*	*Serranidae*	[13]
		*Epinephelus akaara*	*Epinephelidae*	[14]
		*Monopterus albus*	*Synbranchidae*	
		*Thalassoma bifasciatum*	*Labridae*	[15]
		*Centropyge vrolikii*	*Pomacanthidae*	[16]
	both directions serially	*Gobiodon erythrospilus*	*Gobiidae*	[17]
		*Lythyrpnus dalli*	*Gobiidae*	[18]
		*Trimma yanagitai*	*Gobiidae*	[19]
		*Kryptolebias marmoratus*	*Cyprinodontidae*	[14]
gonochoric		*Oreochromis niloticus*	*Cichlidae*	[7]
		*Scatophagus argus*	*Scatophagidae*	[20]
		*Paralichthys olivaceus*	*Paralichthyidae*	[21]
unisexual reproduction		*Poecilia formosa*	*Cyprinodontidae*	[22]

**Table 2:** Sex-determining systems and sex-determining genes in some scleractinian fish.

Sex-Determining Systems	Exemplar Species	Sex-Determining Genes	Families	Reference
XX/XY	*Oreochromis niloticus*	*AmhY*	*Cichlidae*	[35]
	*Oryzias luzonensis*	*GsdfY*	*Adrianichthyidae*	[36]
	*Oryzias latipes*	*DmY*	*Adrianichthyidae*	[36]
	*Ictalurus punctatus*	*Bcar1*	*Ictaluridae*	[37]
	*Oryzias dancena*	*Sox3Y*	*Adrianichthyidae*	[98]
	*Oncorhynchus mykiss*	*SdY*	*Salmonidae*	[99]
	*Nothobranchius furzeri*	*Gdf6Y*	*Nothobranchiidae*	[100]
	*Plecoglossus altivelis*	*AmhIIbY*	*Plecoglossidae*	[101]
	*Gadusmorhua*	*ZkY*	*Gadidae*	[102]
	*Arapaima gigas*	*Id2bbY*	*Osteoglossidae*	[103]
	*Clupea harengus*	*Bmpr1bbY*	*Clupeidae*	[104]
	*Silurus lanzhouensis*	*AmhrIIY*	*Siluridae*	[61]
	*Silurus meridionalis*	*AmhrIIY*	*Siluridae*	[62]
ZZ/ZW	*Cynoglossus semilaevis*	*Dmrt1*	*Cynoglossidae*	[38]
	*Oreochromis aureus*	*Banf-W*	*Cichlidae*	[105]
	*Seriola quinqueradiata*	*Hsd17b1*	*Carangidae*	[44]
	*Scophthalmus maximus*	*Sox2*	*Scophthalmidae*	[106]
X_1_X_1_X_2_X_2_/X_1_X_2_Y	*Collichthys lucidus*	*Dmrt1*	*Sciaenidae*	[107]

## Data Availability

The data presented in this study are available in this article.
